# Role of endoscopic ultrasonography for differential diagnosis of upper gastrointestinal submucosal lesions

**DOI:** 10.1186/s12876-021-01945-9

**Published:** 2021-10-07

**Authors:** Qian Su, Jin Peng, Xiong Chen, Zhiming Xiao, Rui Liu, Fen Wang

**Affiliations:** 1 Department of Gastroenterology, Hunan Key Laboratory of Non-Resolving Inflammation and Cancer, The Third Xiangya Hospital of Central South University, 138 Tongzi Road, Changsha, 410013 Hunan People’s Republic of China; 2 First Hospital of Changsha, 311 Yinpan Road, Changsha, 410005 Hunan People’s Republic of China

**Keywords:** Endoscopic ultrasonography, Upper gastrointestinal submucosal lesion, Stromal tumor, Leiomyoma, Invasive risk

## Abstract

**Objective:**

To determine the accuracy of endoscopic ultrasonography (EUS) in the diagnosis of upper gastrointestinal submucosal lesions (SMLs).

**Methods:**

This was a retrospective study involving patients diagnosed with SMLs using EUS and confirmed by histopathology from November 2014 to December 2020 at The Third Xiangya Hospital of Central South University.

**Results:**

A total of 231 patients with SMLs were examined by EUS. Histologically, 107 lesions were stromal tumors, and 75 lesions were leiomyomas. Stromal tumors were mainly located in the stomach (89.7%), and leiomyomas were predominantly seen in the esophagus (69.3%). The diagnostic accuracy of EUS for stromal tumors and leiomyomas was 80.4% and 68.0%, respectively. The diagnostic accuracy was highest for lesions located in the muscularis mucosa. The mean diameter of stromal tumors measured using EUS was significantly larger than that of leiomyomas (21.89 mm vs. 12.35 mm, *p* < 0.001). Stromal tumors and leiomyomas originated mainly from the muscularis propria (94.4%) and the muscularis mucosa (56.0%), respectively. Compared with the very low-risk and low-risk groups of stromal tumors according to the National Institute of Health guidelines, the intermediate-risk and high-risk groups were more likely to have a lesion > 3 cm (*p* < 0.001) and a surface ulcer (*p* < 0.01) identified by EUS.

**Conclusions:**

EUS has good diagnostic value for the diagnosis of upper gastrointestinal SMLs based on the lesion size and the muscle layer of origin. The diagnostic accuracy of EUS lesions is related to the origin, and the diagnostic accuracy is greatest in the mucosal muscularis layer. Stromal tumors > 3 cm and a surface ulcer on EUS are likely to be intermediate or high risk for invasion.

## Introduction

Submucosal lesions (SMLs) in the digestive tract refer to abnormal lesions originating from the various layers under the mucosa, and upper gastrointestinal SMLs are the most common. Differential diagnosis of the various types of SMLs during routine endoscopy is difficult [1]. Different pathological types of SMLs have various biological behaviors. Pathologically, they can be divided into benign, malignant, and potentially malignant categories. Therefore, it is important to distinguish the different pathological types of upper gastrointestinal SMLs. Endoscopic ultrasonography (EUS) is a very useful tool for the evaluation of these upper gastrointestinal SMLs. Not only can it be used to observe the morphological structure of the lesions in the cavity, but EUS can also perform real-time scanning to obtain information regarding the wall layer structure of the digestive tract, the originating layer of the lesions, and the relationship between the extent of lesion infiltration and the surrounding tissues, peripheral lymph nodes and adjacent organs. EUS-guided fine-needle aspiration/biopsy (EUS-FNA/FNB) can further help to obtain cells or tissues for pathological confirmation [2, 3, 7]. In this study, we reviewed and compared the distribution, size, endoscopic features, and pathological results of SMLs to determine the diagnostic value of EUS for SMLs. This was performed by analyzing the diagnostic consistency rate, the relationship between endoscopic features and invasion risk, and the relationships among the distribution, diameter, origin and pathological properties of the lesions.

## Materials and methods

### Patient selection

In this retrospective study, adult patients with SMLs diagnosed and treated at The Third Xiangya Hospital of Central South University from November 2014 to December 2020 were included and their clinical data were retrieved. The study was approved by the Ethics Committee of The Third Xiangya Hospital of Central South University. All patients gave written informed consent for the endoscopic and surgical procedures. Patients were excluded if they were under 18 years of age, had incomplete endoscopic or pathological data, or were pregnant.

All patients underwent a routine endoscopy and EUS to confirm the location of the SML in the upper gastrointestinal tract. Six patients underwent EUS-FNA/FNB prior to surgery, 14 patients underwent EUS elastography, and one patient underwent contrast-enhanced EUS. Meanwhile, all patients underwent an endoscopic or surgical resection. Postoperative specimens were collected for pathological and immunohistochemical examination.

### Instruments and procedures

After eight hours of fasting before the procedure and obtaining written informed consent, oral gel was applied for local anesthesia and intravenous propofol was administered for sedation. A mini-probe (20 MHz; SP-701, Fujiron, Tokyo, Japan) or linear array ultrasound probe (6.0–7.5 MHz; EU-ME2, Olympus, Tokyo, Japan) was selectively used according to the findings of the routine endoscopy. The lumen was filled with water for scanning. Once the position of the lesion was identified, the size was measured, and the origin and echo characteristics of the lesion were determined (heterogeneity, whether the boundary was clear, etc.). The ultrasound device was then used in Doppler mode to detect blood flow, velocity, and direction.

### Endoscopic ultrasound stratification of the normal digestive tract wall

According to a study by Kuroki et al. [4], the digestive tract wall can be divided into five layers by EUS. By comparing the results with histological findings, the first hyperechoic layer was identified as the mucosal reflex interface, the second hypoechoic layer was the muscularis mucosa, the third hyperechoic layer was the submucosal layer, the fourth hypoechoic layer was the muscularis propria, and the fifth hyperechoic layer was the serosal layer. The upper and middle third esophageal wall did not have a serosal layer, and the fifth layer was the surrounding connective tissue.

### Determination of invasive risk by stromal tumors

According to the revised recommendations of the National Institute of Health (NIH) standard titled "Consensus for the Diagnosis and Treatment of Gastrointestinal Stromal Tumors in China (2017 Edition)" [5], patients with stromal tumors were divided into one of four groups based on the risk of tumor invasion, including very low-risk (VLR), low-risk (LR), intermediate-risk (IR) and high-risk (HR).

### Statistical analysis

Statistical analyses were performed with SPSS version 25.0 (IBM). Quantitative data were shown as mean ± standard deviation (SD) and were compared using the *t*-test or Mann–Whitney *U* test. The chi-square test or Fisher’s exact test were used to compare categorical data. A *p* value less than 0.05 was considered statistically significant.

## Results

### Clinical data

**Fig. 1:**
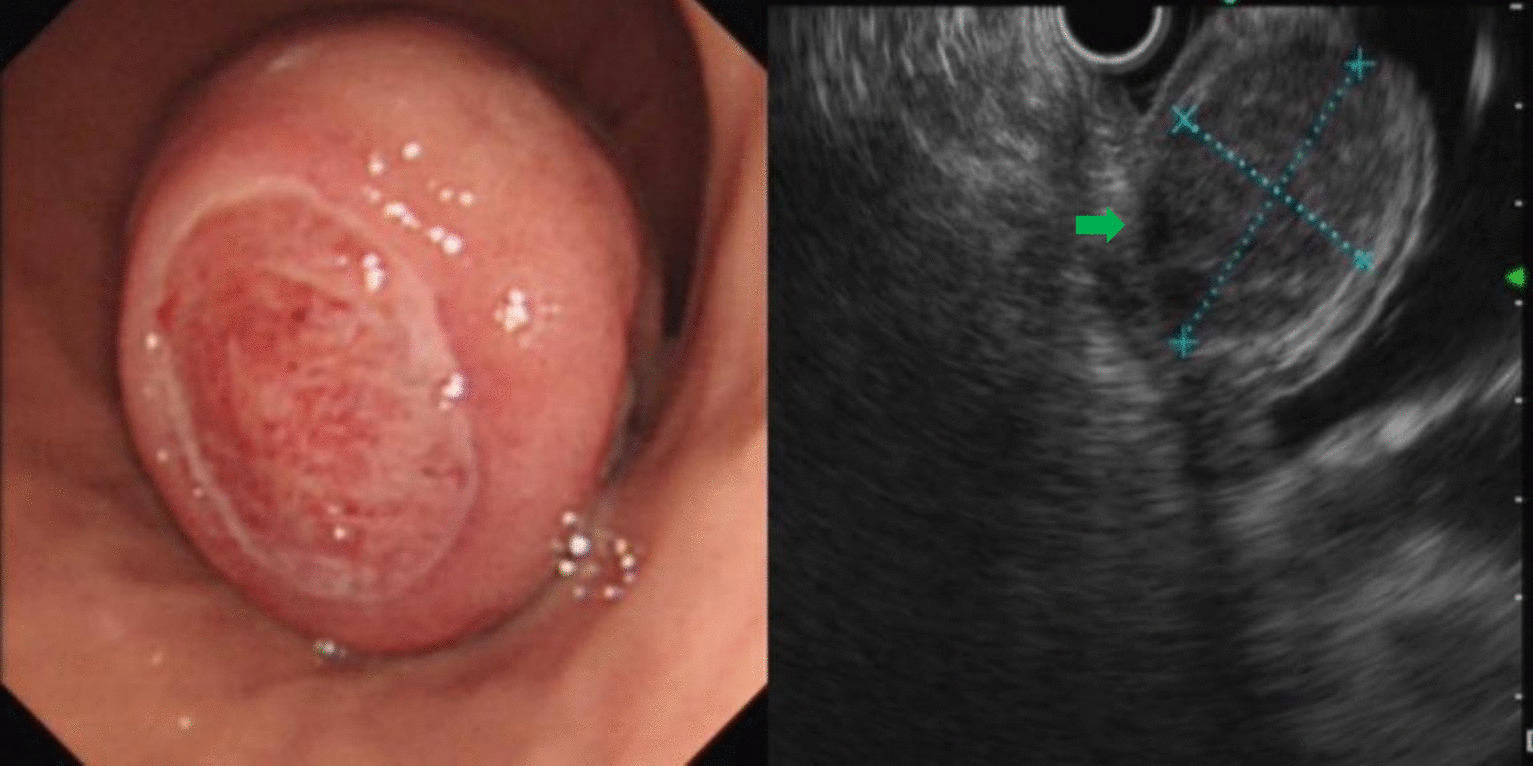
Stromal tumor of gastric body (green arrow)

**Fig. 2:**
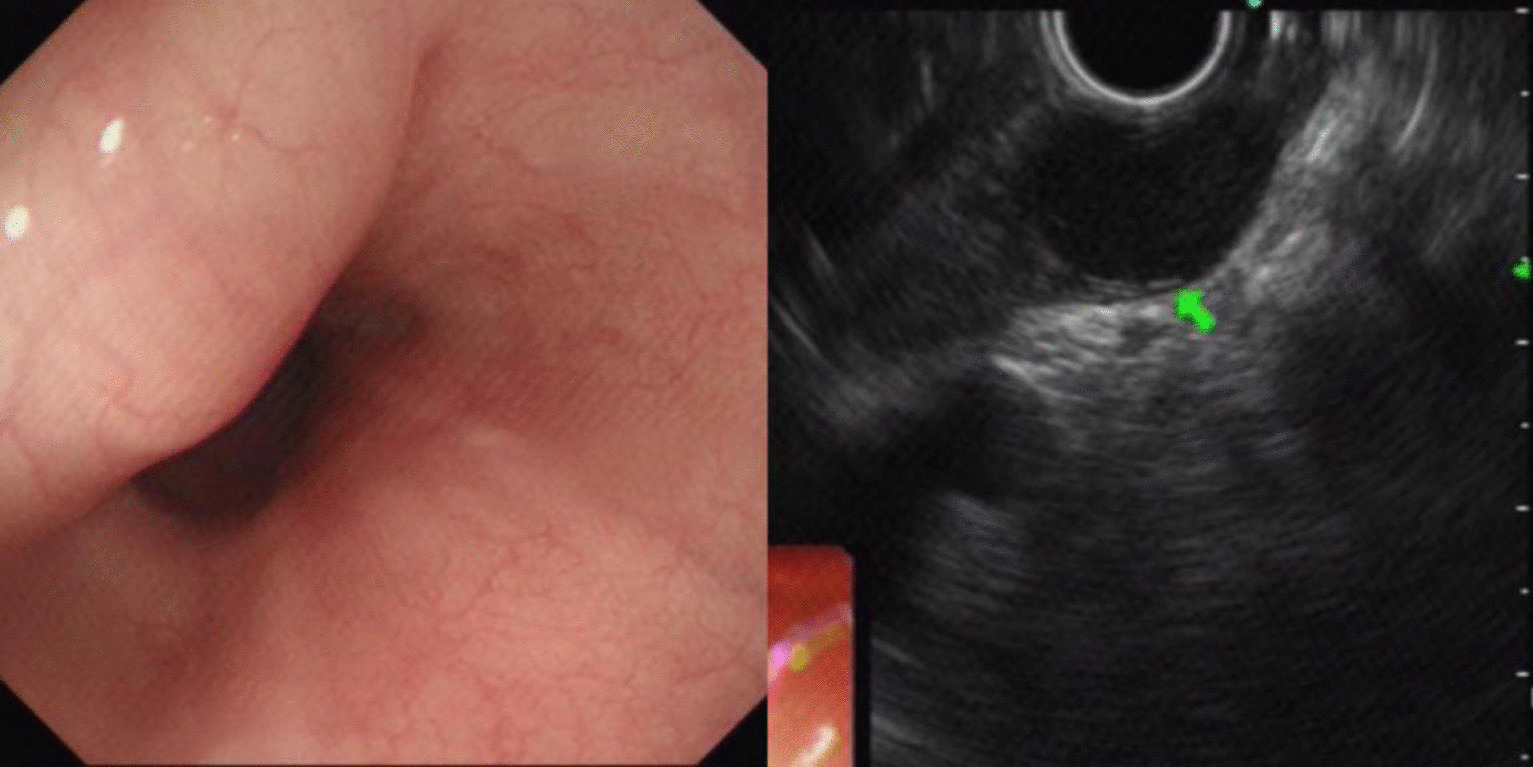
Leiomyoma of esophagus (green arrow)

**Fig. 3:**
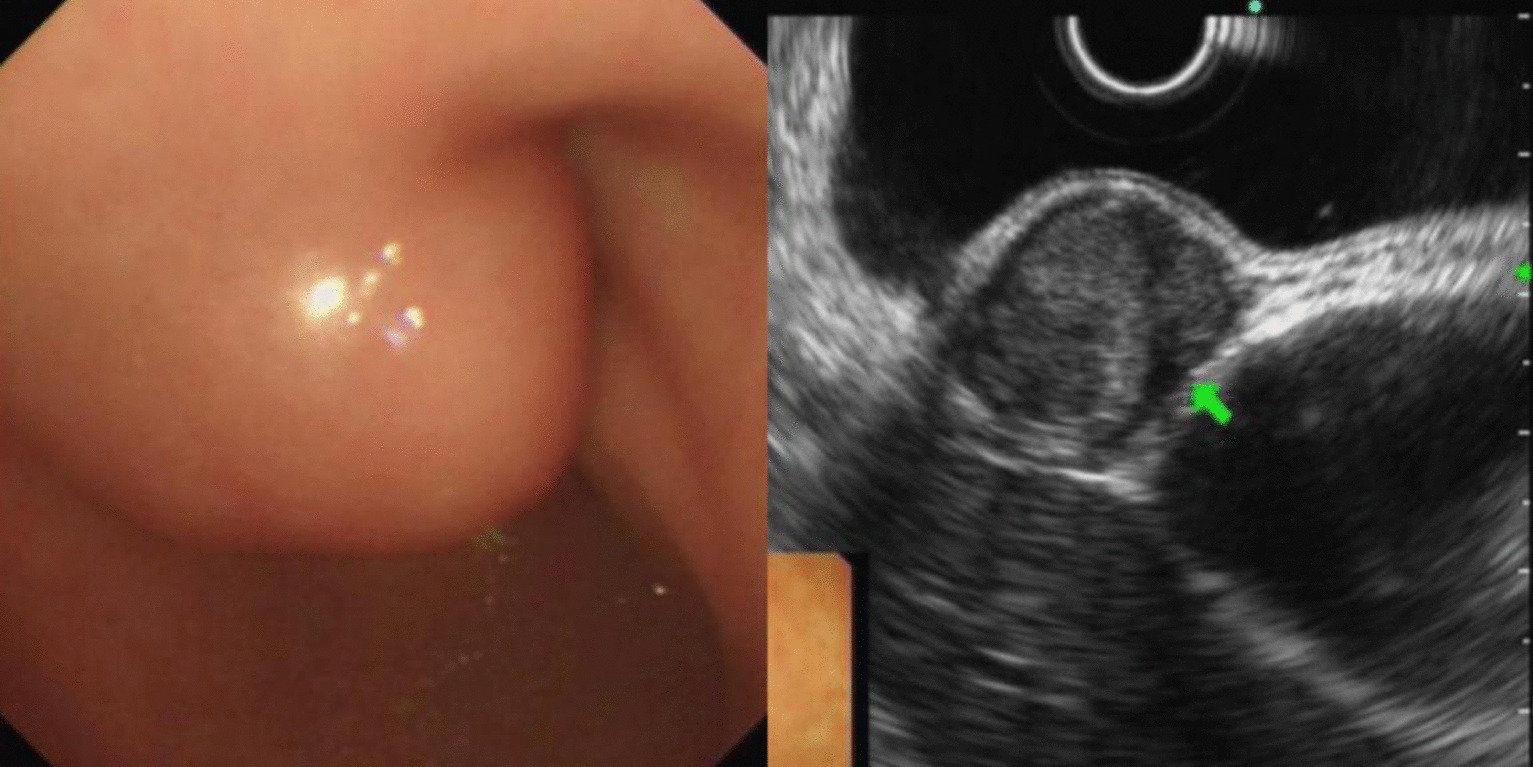
Schwannoma of gastric body (green arrow)

**Fig. 4:**
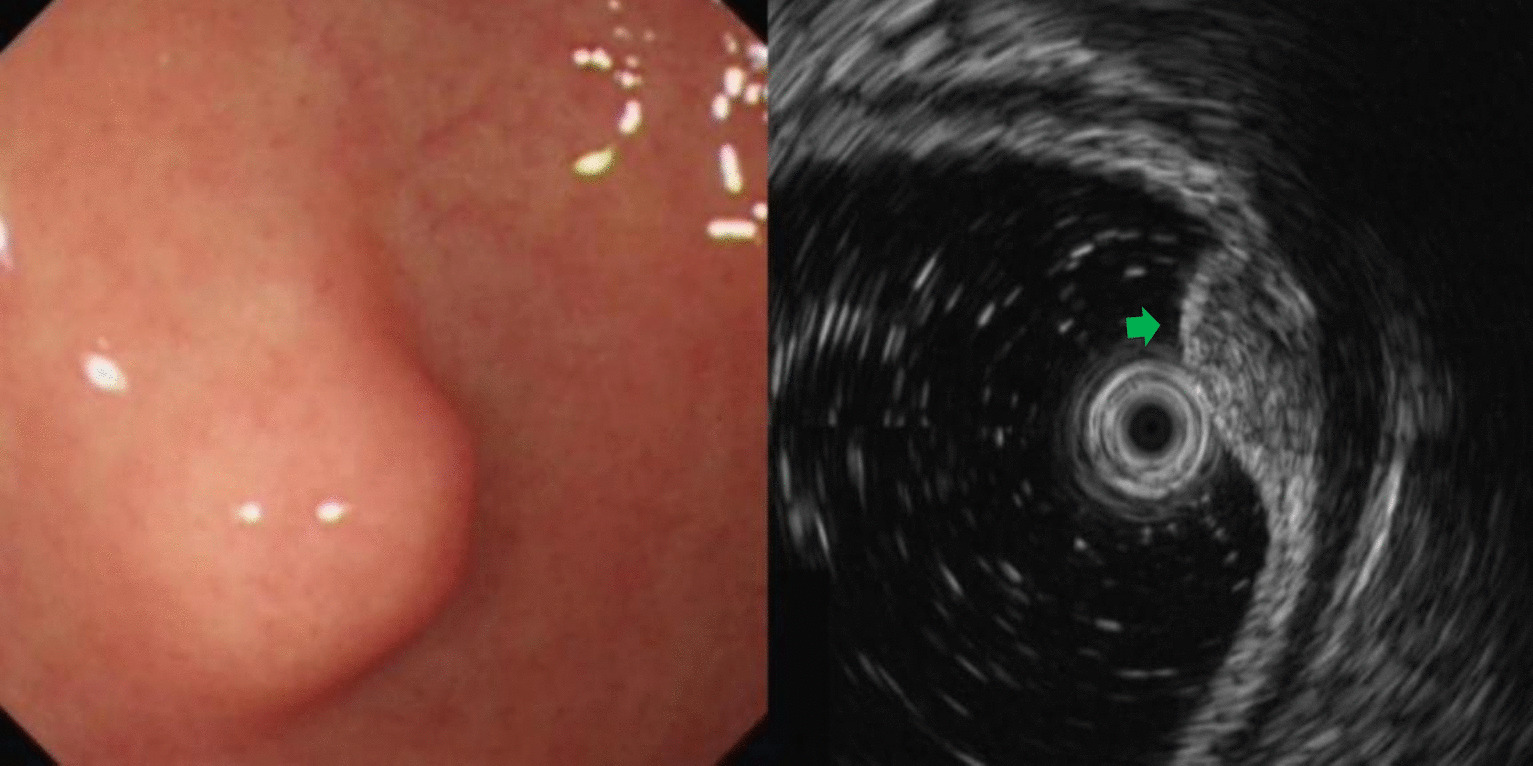
Heterotopic pancreas of gastric antrum (green arrow)

**Fig. 5:**
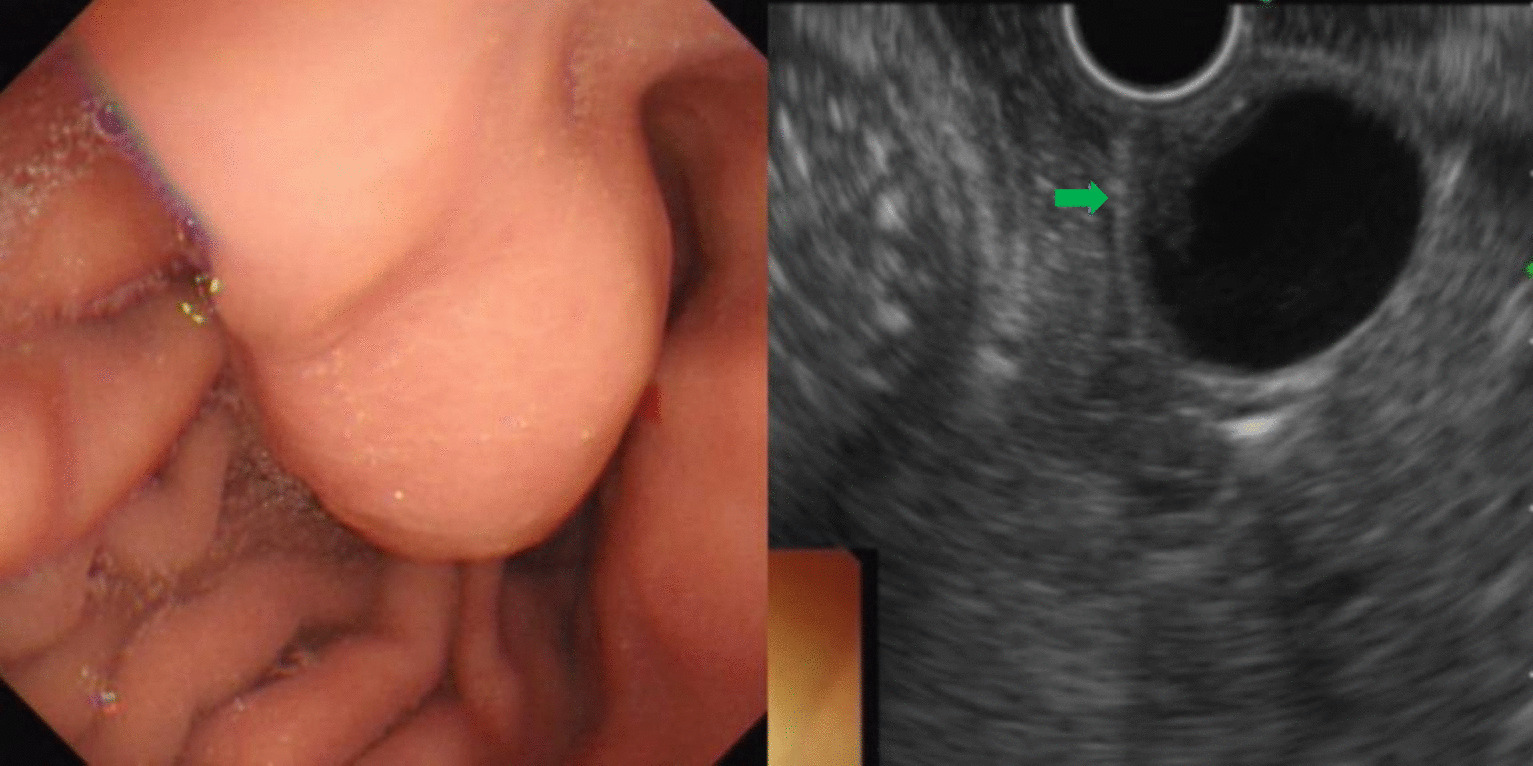
Benign cyst of gastric body (green arrow)

**Fig. 6:**
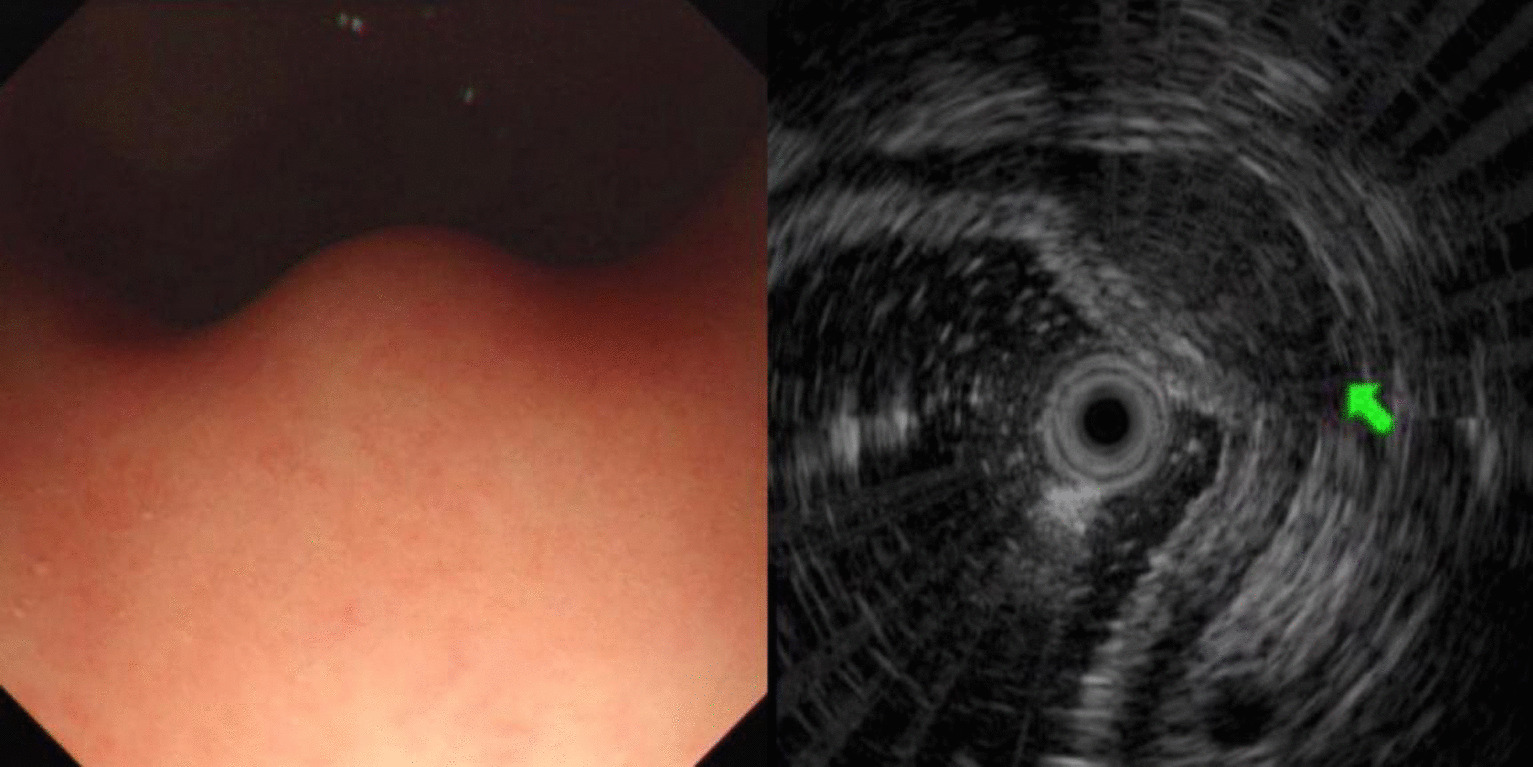
Glomus tumor of gastric antrum (green arrow)

**Fig. 7:**
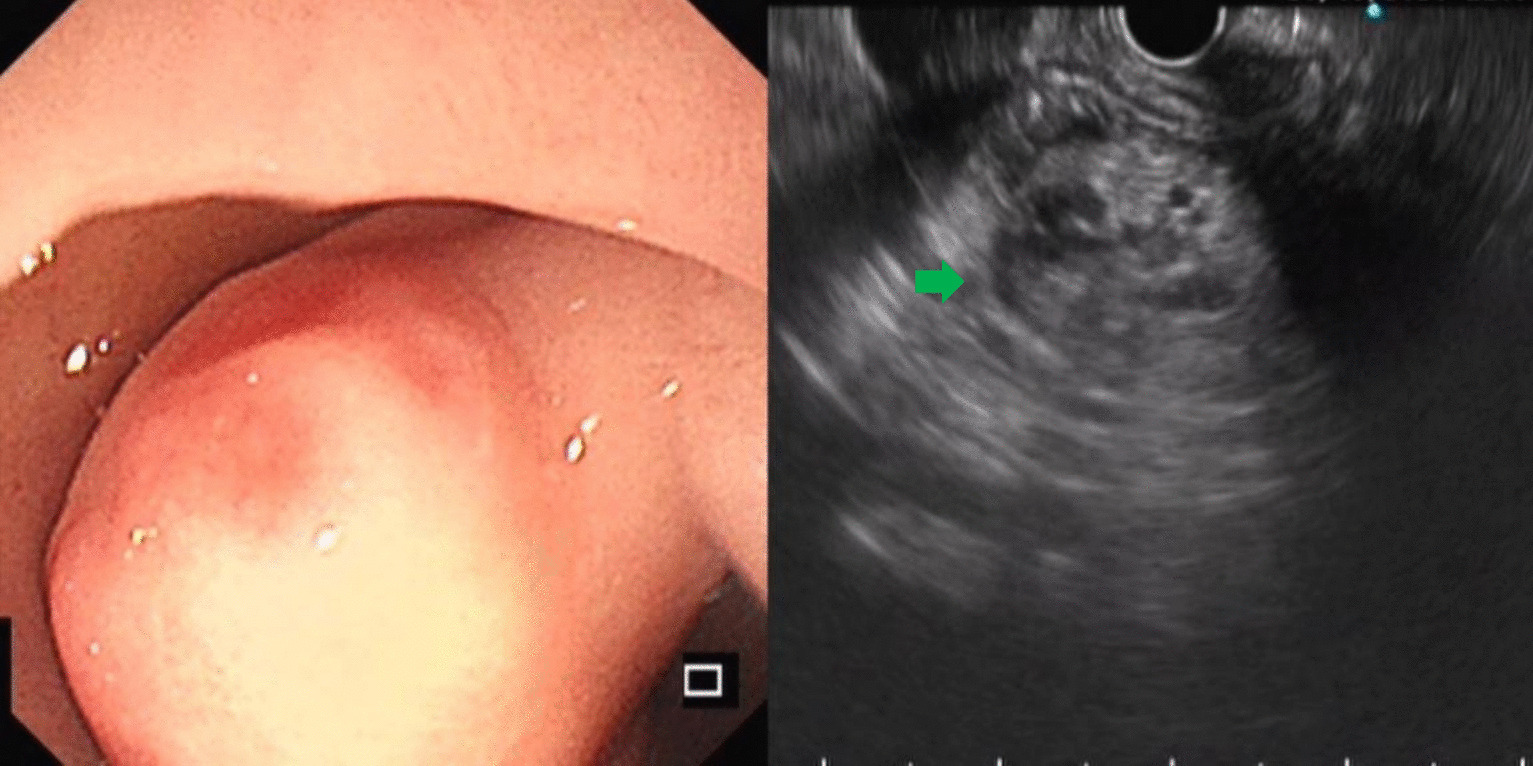
Hamartoma of duodenal bulb (green arrow)

**Fig. 8:**
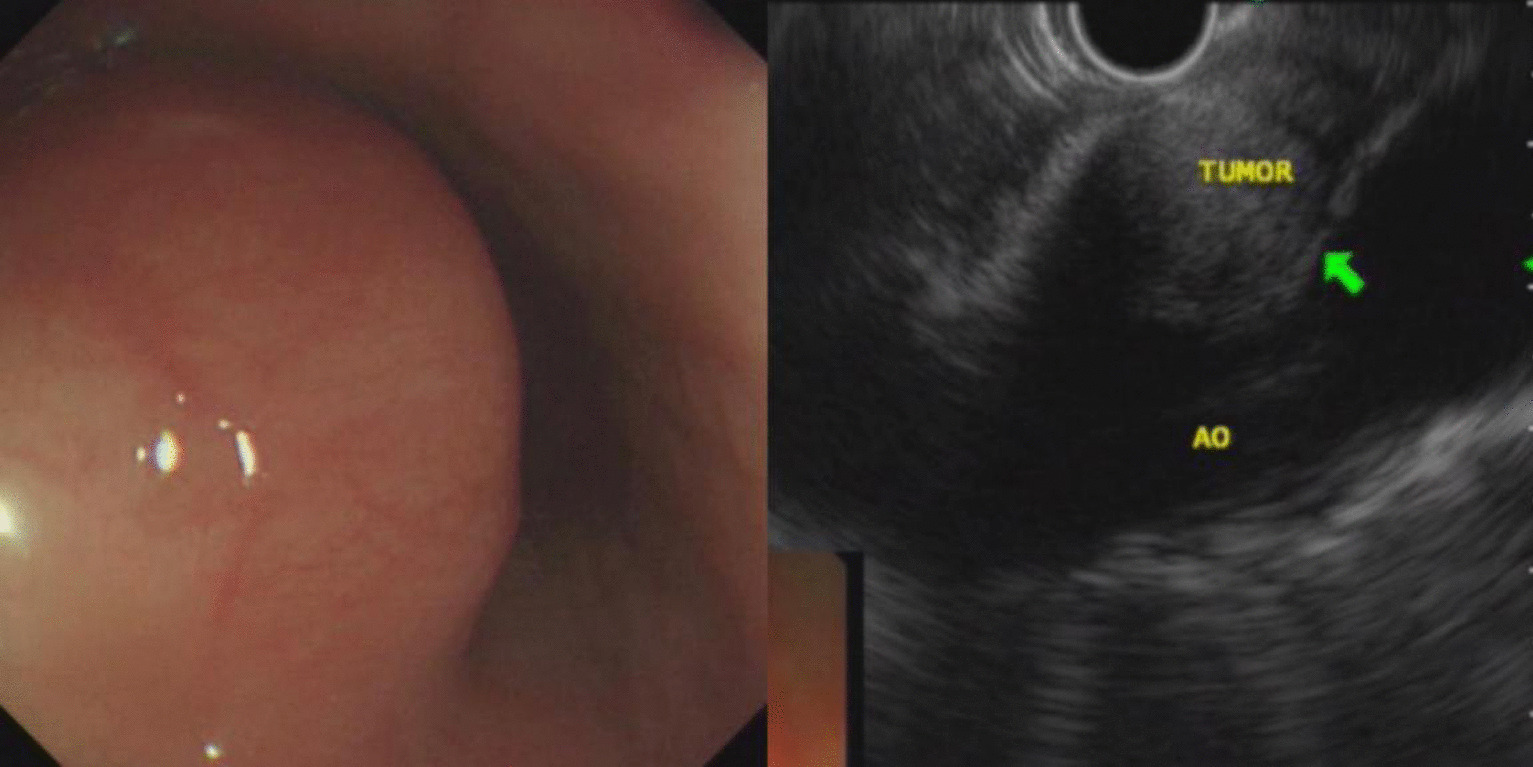
Solitary fibroma of esophagus (green arrow)

**Fig. 9:**
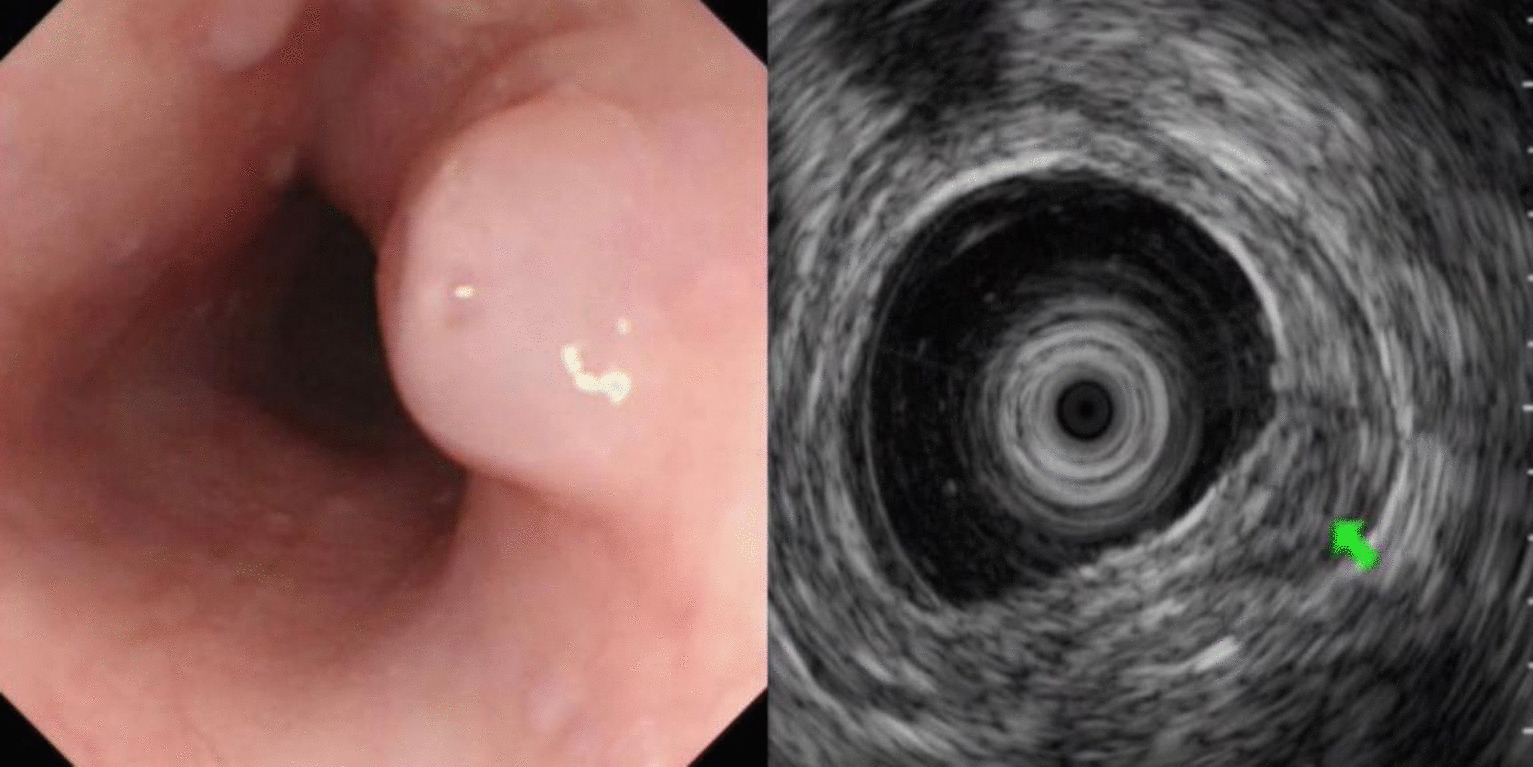
Haemo-lymphangioma of esophagus (green arrow)

**Fig. 10:**
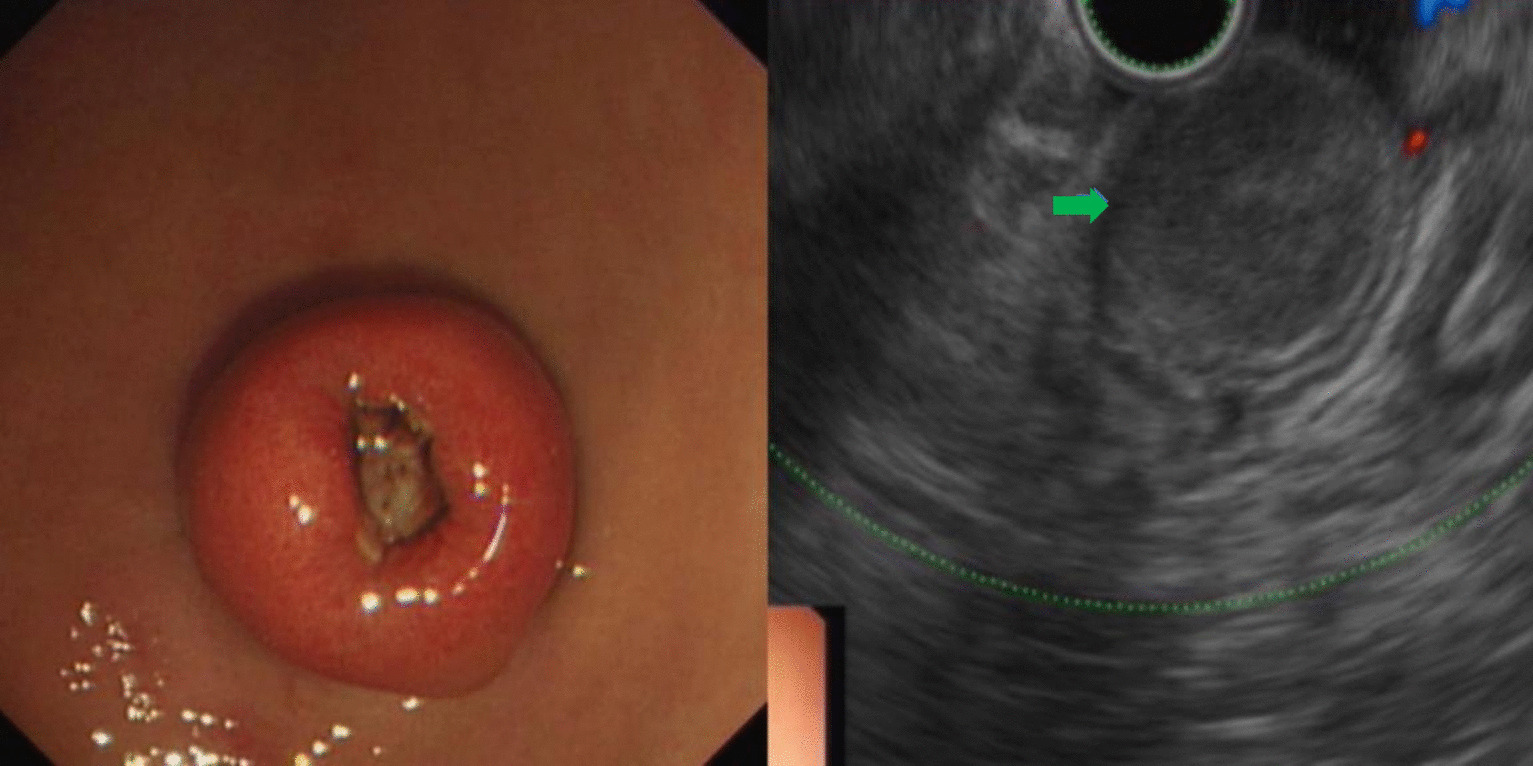
Angiogenic tumor of gastric antrum (green arrow)

A total of 231 patients with SMLs diagnosed during the study period were included for analysis. There were 122 males and 109 females with a mean age of 51.89 ± 11.91 years (range: 22–79 years). The most common site of esophageal lesions was the upper and middle third of the esophagus while that of the stomach was the gastric fundus (Table 1). The most common histological types of SMLs in the esophagus and stomach were leiomyomas and stromal tumors, respectively (Table 2; Figs. 1, 2). The other SMLs observed in the study patients were schwannoma (Fig. 3), heterotopic pancreas (Fig. 4), benign cyst (Fig. 5), glomus tumor (Fig. 6), hamartoma (Fig. 7), solitary fibroma (Fig. 8), haemo-lymphangioma (Fig. 9) and angiogenic tumor (Fig. 10). The most common sites of origin of stromal tumors, leiomyomas and ectopic pancreas were the muscularis propria, muscularis mucosa, and submucosa, respectively (Table 3).

**Table 1:** Distribution of lesions

Origin	Number of cases	Percentage
*Esophagus*	63	27.3
Upper esophagus	23	10.0
Mid-esophagus	22	9.5
Lower esophagus	15	6.5
Multiple locations	3	1.3
*Stomach*	159	68.8
Gastric angle	3	1.3
Junction between antrum and body	4	1.7
Cardia	5	2.2
Junction between fundus and body	6	2.6
Gastric antrum	14	6.1
Gastric body	45	19.5
Gastric fundus	82	35.5
*Duodenum*	9	3.9
Descending duodenum	5	2.2
Duodenal bulb	4	1.7
Total	231	100

**Table 2:** Distributions of submucosal lesions (SMLs) in the upper digestive tract based on histopathology

Pathological result	Esophagus	Stomach	Duodenum	Total	Percentage	*p*
Stromal tumor	6	96	5	107	46.3	0.001**
Leiomyoma	52	22	1	75	32.5	0.001**
Heterotopic pancreas	–	11	2	13	5.6	0.011*
Schwannoma	–	11	–	11	4.8	0.080
Inflammation	1	8	1	10	4.3	0.222
Benign cyst	2	2	–	4	1.7	0.416
Glomus tumor	–	4	–	4	1.7	0.640
Angiolipoma	–	3	–	3	1.3	0.606
Hamartoma	–	1	–	1	0.4	1.000
Solitary fibroma	1	–	–	1	0.4	0.311
Haemo-lymphangioma	1	–	–	1	0.4	0.311
Angiogenic tumor	–	1	–	1	0.4	1.000
Total	63	159	9	231	100	

*, **Esophagus, the stomach, and the duodenum have significant differences at the levels of *p* < 0.05, *p* < 0.01, respectively

**Table 3:** Originating layers of the lesions on endoscopic ultrasonography (EUS)

Pathological results	Muscularis mucosa layer	Submucosal layer	Muscularis propria layer	Total	*p*
Stromal tumor	6	–	101	107	0.001**
Leiomyoma	42	–	33	75	0.001**
Heterotopic pancreas	1	11	1	13	0.001**
Schwannoma	–	1	10	11	0.094
Inflammation	3	–	7	10	0.836
Benign cyst	1	–	3	4	1.000
Glomus tumor	–	–	4	4	0.663
Angiolipoma	–	–	3	3	1.000
Hamartoma	–	–	1	1	1.000
Solitary fibroma	–	–	1	1	1.000
Haemo-lymphangioma	1	–	–	1	1.000
Angiogenic tumor	–	–	1	1	1.000
Total	54	12	165	231	

**Mmuscularis mucosa layer, the submucosal layer, and the muscularis propria layer have significant differences at the levels of *p* < 0.01

### Diagnostic accuracy of EUS

The diagnostic accuracy of EUS for stromal tumors was 80.4% and for leiomyomas was 68.0% (Table 4). The data were divided into two groups: (A) the EUS diagnosis and pathological diagnosis were the same, and (B) the diagnoses differed. Differences regarding patient age, gender, lesion diameter, location, and origin were compared between the two groups. The results showed that the difference in lesion origin was statistically significant (*p* < 0.05). Furthermore, the diagnostic accuracy of EUS was highest for the lesions located in the muscularis mucosa (muscularis mucosa vs. muscularis propria *p* < 0.001; muscularis mucosa vs. submucosa *p* < 0.001; muscularis propria vs. submucosa *p* = 0.001; Table 5).

**Table 4:** Diagnostic rates of endoscopic ultrasonography (EUS) diagnosis based on final histopathology of the lesions

Pathological result	Number of pathological diagnoses	Number of EUS diagnoses	Number of consistent diagnoses between pathology and EUS	Diagnostic consistency rate
Stromal tumor	107	147	86	80.4%
Leiomyoma	75	82	51	68.0%
Heterotopic pancreas	13	1	1	7.7%
Schwannoma	11	–	–	0
Inflammation	10	–	–	0
Benign cyst	4	–	–	0
Glomus tumor	4	–	–	0
Angiolipoma	3	–	–	0
Hamartoma	1	–	–	0
Solitary fibroma	1	–	–	0
Haemo-lymphangioma	1	–	–	0
Angiogenic tumor	1	–	–	0
Total	231	231	138	59.7%

**Table 5:** Comparison of diagnosis by endoscopic ultrasonography (EUS) with pathological diagnosis

	Comparison of diagnosis on EUS with pathological diagnosis		*p*
	Consistent (n = 138)	Not consistent (n = 93)	
Age	53.67 ± 11.47	49.29 ± 11.99	0.407
Mean diameter of lesions measured by EUS	17.83 ± 14.68	18.25 ± 13.49	0.823
Mean diameter of lesions based on measurement of surgical specimens	23.25 ± 19.05	19.69 ± 14.13	0.152
Gender			
Male	78	44	0.169
Female	60	49	
Distribution			
Esophagus	47	16	0.105
Stomach	86	73	
Duodenum	5	4	
Origin			
Muscularis mucosa layer	45	9	0.000***
Submucosal layer	1	11	
Muscularis propria layer	92	73	

***Consistent diagnosis group and the inconsistent diagnosis group have significant differences at the level of *p* < 0.001

### Comparison of the maximum diameters of stromal tumors and leiomyomas

The mean diameter of the stromal tumors as measured by EUS was 21.89 ± 14.81 mm (range: 3.80–76.10 mm), while that of the leiomyomas was 12.35 ± 11.18 mm (range: 2.30–62.00 mm) and the difference was statistically significant (*p* < 0.001). The mean diameter of stromal tumors based on the measurement of the surgical specimens was 25.49 ± 19.49 mm (range: 5.00–135.00 mm), while that of the leiomyomas was 16.65 ± 13.48 mm (range: 2.00–60.00 mm; *p* < 0.01).

### Comparison of the originating layers of stromal tumors and leiomyomas on EUS

Most of the stromal tumors originated from the muscularis propria (101/107, 94.4%), while the leiomyomas mainly originated from the muscularis mucosa (42/75, 56.0%). This difference was statistically significant (χ^2^ = 57.66, *p* < 0.001).

### Sensitivity, specificity and predictive value of the originating layer on EUS to determine the pathological nature of the submucosal lesions

When the originating layer (muscularis propria) of the lesions was used to make a stromal tumor diagnosis, the sensitivity was 94.4%, specificity was 56.0%, positive predictive value was 75.4% and negative predictive value was 87.5%. Similarly, the sensitivity, specificity, positive predictive value and negative predictive value of the originating layer (muscularis mucosa) of the lesions to make the diagnosis of leiomyoma was 56.0%, 94.4%, 87.5% and 75.4%, respectively.

### Comparison of the EUS characteristics of stromal tumors with different invasive risks

One-hundred-and-seven patients with stromal tumors were categorized into either the VLR group (62 patients), LR group (34 patients), IR group (10 patients) or HR group (one patient) according to the revised NIH standards [5]. To compare characteristics, the patients were further grouped into two groups: the VLR + LR group or the IR + HR group. Compared to the VLR + LR group, the IR + HR group was more likely to have a lesion diameter > 3 cm (*p* < 0.001) as well as a surface ulcer (*p* < 0.01) identified by EUS (Table 6).

**Table 6:** Comparison of the endoscopic ultrasonography (EUS) characteristics of stromal tumors and their categorization into one of two groups based on the risk of invasion

EUS characteristic	VLR + LR (n = 96)	IR + HR (n = 11)	*p*
Diameter measured under EUS			
≤ 3 cm	75	1	0.000***
> 3 cm	21	10	
Surface ulcer			
Yes	17	7	0.002**
No	79	4	
Clear boundary			
Yes	63	8	0.747
No	33	3	
Regular shape			
Yes	46	4	0.467
No	50	7	
Echo intensity			
Low	92	10	0.425
Not low	4	1	
Echo heterogeneity			
Yes	27	1	0.282
No	69	10	

VLR, very low risk; LR, low risk; IR, intermediate risk; HR, high risk

** and ***VLR + LR group and the IR + HR group have significant differences at the levels of *p* < 0.01 and *p* < 0.001, respectively

## Discussion

SMLs have similar appearances on routine endoscopy irrespective of their histology. Moreover, endoscopic biopsy of SMLs is difficult since a routine endoscopy can only visualize and obtain the normal mucosa on the surface of the lesions. Other imaging methods, such as ultrasound, barium meals, and CT, have low sensitivity and specificity for the diagnosis of such lesions [6]. EUS can visualize SMLs of the upper digestive tract and provide information regarding the layered structure of the digestive tract wall, the originating layer of the lesions, and the relationship between the lesion and the surrounding tissues, the peripheral lymph nodes and adjacent organs. Moreover, EUS-FNA/FNB can further help to obtain cells or tissues for pathology [2, 3, 7]. Therefore, EUS has become the most effective method for the diagnosis of SML.

In this study, SMLs were found in the stomach (159 patients), esophagus (63 patients), and duodenum (9 patients). The final pathology suggested that stromal tumors and leiomyomas were the most common types of SMLs, consistent with the report by Dias et al*.* [3]. Stromal tumors were more common in the stomach, while leiomyomas were more common in the esophagus, which is also consistent with previous reports [8].

The postoperative pathological diagnosis was considered the gold standard in this study and was used for comparison with the EUS findings. We found that EUS had good diagnostic value for SMLs. The diagnostic consistency rate of EUS was 80.4% for stromal tumors and 68.0% for leiomyomas, similar to previous studies [9]. The rare tumors that were observed, such as glomus tumors and angiolipomas, lacked characteristic features on EUS and appeared similar to common SMLs, such as stromal tumors and leiomyomas, and may be easily misdiagnosed on EUS. Moreover, the accuracy of EUS diagnosis is closely related to the operator's technological and clinical experience, available EUS equipment and the degree of patient cooperation [10]. Therefore, the diagnosis of such diseases needs to be combined with the patient's medical history and other relevant tests, especially pathological examinations. The diagnostic accuracy of lesion type was related to the level of origin, and the diagnostic accuracy of lesions originating from the muscularis mucosa was the greatest, which was consistent with the report by Schulz et al*.* [11]. This may be due to the many types of submucosal lesions derived from the submucosa and muscularis propria, such as schwannomas, lipomas, ectopic pancreas, and other relatively rare lesions, resulting in insufficient diagnosis experience. Therefore, EUS is reliable for the diagnosis of lesions originating from the muscularis mucosa. For lesions originating from the submucosa or muscularis propria, other auxiliary examinations should be considered or pathology should be obtained for a histological diagnosis.

Additionally, the maximum diameters of the lesions were compared and the results suggested that the mean maximum diameter of stromal tumors was significantly greater than that of leiomyomas. This may provide a reference for differentiation between the two types of SMLs. Moreover, we calculated the sensitivity, specificity, positive predictive value and negative predictive value in distinguishing these two diseases according to the originating layer of the tumor. The results showed that if the lesion was originating from the muscularis propria, then the lesion was more likely to be a stromal tumor, but if the lesion originated from the muscularis mucosa, it was more likely to be a leiomyoma. Therefore, the differential diagnosis of leiomyoma or stromal tumor could be obtained according to the originating layer of the lesion as identified by EUS.

In this study, stromal tumors mostly appeared as hypoechoic masses originating from the muscularis propria (Fig. 1). It is currently believed that all stromal tumors have malignant potential and differentiation between benign and malignant tumors cannot be made easily [12]. The risk of invasion by stromal tumors has been determined based on parameters such as location, size, mitosis rate and whether the tumor has ruptured [5]. Brand et al*.* [13] reported that a heterogeneous echotexture greater than 3 cm and with irregular margins may suggest higher-risk stromal tumors. A study by Jeon and colleagues [14] found that the presence of mucosal ulceration suggests a higher risk of malignancy. We compared the EUS characteristics of stromal tumors between the VLR + LR and the IR + HR groups, and found that stromal tumors with a diameter greater than 3 cm or a surface ulcer on EUS were more likely to be of intermediate or high risk.

Leiomyomas were found to originate primarily in the muscularis mucosa. They appeared as homogeneous or non-homogeneous hypoechoic masses with clear boundaries on EUS, similar to the findings of Codipilly et al. [15] (Fig. 2). A study by Park and colleagues [16] found that 71% of gastric schwannomas exhibited echogenicity similar to the muscularis propria. In this study, schwannomas mostly manifested as heterogeneous hypoechoic or mixed-echogenic masses originating from the muscularis propria, with clear boundaries and poor blood flow signals (Fig. 3). Chen et al. [17] reported that the EUS features of heterotopic pancreas include indistinct margins, heterogeneous echogenicity (mainly hypoechoic accompanied by small, scattered hyperechoic areas), and duct-like structures with or without echoes. In our study, the heterotopic pancreases were mostly heterogeneous, mixed-to-low-echogenic masses originating from the submucosal layer, and occasionally presented with a luminal-like structure (Fig. 4). Other rare SMLs found in this study included benign cysts (Fig. 5), glomus tumors (Fig. 6), hamartoma (Fig. 7), solitary fibromas (Fig. 8), haemo-lymphangioma (Fig. 9), angiogenic tumor (Fig. 10) and angiolipoma. Multicenter studies with larger sample sizes are required to analyze the EUS characteristics of these uncommon lesions.

This study has some limitations. Firstly, it was a single-center, retrospective study. Secondly, the role of EUS-FNA/FNB, elastography, or contrast-enhanced EUS in the differentiation between leiomyoma and stromal tumors could not be determined due to the small number of cases. Currently, there are few reports on the role of EUS elastography in the diagnosis of SML [18, 19]. Several clinical studies have reported the diagnostic value of contrast-enhanced EUS for SML [20, 21]. One study by Kamata et al. showed that hyper-enhancement and inhomogeneous enhancement were found to be characteristic features of stromal tumors [22]. In the current study, there were too few cases to draw any meaningful conclusions. Future larger prospective studies are needed for additional analysis.

In conclusion, EUS has good diagnostic value for the diagnosis of upper gastrointestinal SMLs. It may be helpful to identify common SMLs based on the size of the lesion and the originating layer as identified by EUS. Stromal tumors with a diameter larger than 3 cm and a surface ulcer on EUS are more likely to be of intermediate or high risk for malignant potential. EUS is reliable for the diagnosis of lesions from the muscularis mucosa. However, the diagnosis of heterotopic pancreas, inflammation, benign cyst, glomus tumor, hamartoma, solitary fibroma, haemo-lymphangioma, angiogenic tumor and angiolipoma by EUS is more difficult due to the lack of characteristic features and rarity of these diseases.

## Data Availability

All other data used to generate tables for the study are available upon request by the corresponding author.
